# LATER-LIFE TRAJECTORIES OF MARITAL QUALITY FOR WOMEN AND MEN

**DOI:** 10.1093/geroni/igac059.2339

**Published:** 2022-12-20

**Authors:** Jennifer Bulanda, Takashi Yamashita, J Scott Brown

**Affiliations:** Miami University, Oxford, Ohio, United States; University of Maryland, Baltimore County, Baltimore, Maryland, United States; Miami University, Oxford, Ohio, United States

## Abstract

It is essential to better understand older adults’ marital dynamics, as marital quality is consequential for older adults’ physical and mental health, relationships with other social network members, and risk of divorce. Although numerous studies examine marital quality during the early life course, much less is known about later life trajectories. Earlier work suggested marital quality followed a U-shaped curve, characterized by a later-life upswing, but more recent research finds patterns of stability or decline over time. Gender differences in marital quality are well established, but it is less clear whether older men and women experience different marital quality trajectories during later life. We use nationally-representative data from a sample of continuously-married adults over age 50 in the Health and Retirement Study (n=2,175) with measures of both positive and negative marital quality at three time points (2006, 2010, and 2014). Latent growth mixture models in Mplus estimate trajectories of marital quality over the eight-year time period. Results show small declines in both positive (b = -0.016, p < 0.05) and negative (b = -0.028, p < 0.05) marital quality over the observation period, but the declines in positive marital quality are limited to those in remarriages. Women have lower initial positive marital quality and higher initial negative marital quality than men, but there are no significant gender differences in change over time. Results support the stability and continuous-decline patterns of marital quality over the life course rather than a U-shaped curve, and suggest persistence of gender differences over time.

